# The increase in hydric volume is associated to contractile impairment in the calf after the world’s most extreme mountain ultra-marathon

**DOI:** 10.1186/s13728-015-0037-6

**Published:** 2015-10-20

**Authors:** Damien Vitiello, Francis Degache, Jonas J. Saugy, Nicolas Place, Federico Schena, Grégoire P. Millet

**Affiliations:** Faculty of Biology and Medicine, ISSUL, Institute of Sport Sciences, University of Lausanne, Geopolis, 1015 Lausanne, Switzerland; Health Research Unit, School of Health Sciences, University of Applied Sciences Western Switzerland, Lausanne, Switzerland; Department of Physiology, Faculty of Biology and Medicine, University of Lausanne, Lausanne, Switzerland; Faculty of Motor Sciences, University of Verona, Verona, Italy

**Keywords:** Ultra-endurance, Ultra-running, Muscle force loss, Oedema, Inflammation

## Abstract

**Background:**

Studies have recently focused on the effect of running a mountain ultra-marathon (MUM) and their results show muscular inflammation, damage and force loss. However, the link between peripheral oedema and muscle force loss is not really established. We tested the hypothesis that, after a MUM, lower leg muscles’ swelling could be associated with muscle force loss. The knee extensor (KE) and the plantar flexor (PF) muscles’ contractile function was measured by supramaximal electrical stimulations, potentiated low- and high-frequency doublets (PS10 and PS100) of the KE and the PF were measured by transcutaneous electrical nerve stimulation and bioimpedance was used to assess body composition in the runners (*n* = 11) before (Pre) and after (Post) the MUM and compared with the controls (*n* = 8).

**Results:**

The maximal voluntary contraction of the KE and the PF significantly decreased by 20 % Post-MUM in the runners. Hydration of the non-fat mass (NF-Hyd) and extracellular water volume (Ve) were increased by 12 % Post-MUM (*p* < 0.001) in the runners. Calf circumference (+2 %, *p* < 0.05) was also increased. Significant relationships were found for percentage increases in Ve and NF-Hyd with percentage decrease in PS10 of the PF (*r* = −0.68 and *r* = −0.70, *p* < 0.05) and with percentage increase of calf circumference (*r* = 0.72 and *r* = 0.73, *p* < 0.05) in the runners.

**Conclusions:**

The present study suggests that increases in circumference and in hydric volume are associated to contractile impairment in the calf in ultra-marathon runners.

## Background

Mountain ultra-marathons (MUMs) have become increasingly popular in the last decade [1]. Previous studies have investigated the acute consequences of these MUMs on neuromuscular function [2, 3], cardiac function [4], lung function [5, 6], energy [7] and postural control [8]. Changes in biomarkers have been reported after these events [9]. Inflammation and muscular damage have been notably observed after ultra-marathons with large increases in circulating levels of myoglobin, creatine kinase and C-reactive protein [2, 3]. These results confirm that running a MUM is very exhausting for the athlete’s body. However, it was notably demonstrated that MUMs longer than 100 h led to smaller decreases in the maximal voluntary contraction force of the knee extensor (KE) or plantar flexor (PF) muscles than after a MUM <50 h, emphasising the relative muscle force preservation and less muscle damage and inflammation. This difference was previously explained by a specific pacing strategy and the smaller mechanical stress on the muscles of the lower limbs (due to a larger portion of walking vs. running) in longer events [3].

The mechanisms involved in muscle force loss previously observed in athletes after MUMs [2, 3] have remained unclear. Putative relationships might exist because decreases in muscle isometric force and swelling of the sarcoplasmic reticulum were observed in rat muscle fibres after repeated short maximal contractions [10]. To our knowledge, there have been no data regarding the potential relationship between force loss and muscle circumference increase or peripheral oedema after a single-stage MUM >100 h. Interestingly, one study reported not only a muscle force impairment in healthy volunteers after damaging exercise but also a second decrease in muscle strength after 2 days [11]. This second decrease in the maximal voluntary force of the plantar flexors was not related to electromyographic activity reduction but to an increase in muscle thickness, suggesting a direct impact of muscle volume expansion and muscular strength loss. This last finding is important because increased volume of an athlete’s body extremities or limbs and decreased muscle force loss have been reported after ultra-running events. Therefore, muscle volume expansion (i.e. in the muscles of the lower leg) appears to be related potentially to muscle force loss in athletes after MUMs.

Among the mechanisms of muscle volume expansion, an increase in total body water and the development of peripheral oedema have been reported in the context of ultra-marathon running, including a 6 % increase in total body water after a 1200 km run over 17 consecutive days [12]. Increased volumes of athletes’ body extremities (e.g. feet) or limbs (e.g. calf, arm) [13, 14] have also been reported after ultra-running events, and it has been suggested that an increase in total body water might be involved in this fluid shift to distal compartments. That muscle volume expansion mainly occurs in the distal parts of the limbs has been explained by an imbalance between the tone of vasoconstrictor neuron supplying arteries and those supplying veins, which induce impairment of venous return, capillary pressure increases and potentially oedema [15]. These results are in accordance with previous studies demonstrating leg volume increase and ankle oedema after 5–7 consecutive days of hill-walking [16, 17]. Thus, it could be hypothesised that ultra-long duration exercise might be the cause of fluid shifts into extracellular compartments in the distal parts of the runners’ limbs. Oedema can also potentially induce muscle volume expansion, notably in the calf during extreme MUMs. The increase in the internal fluid pressure can lead to a rise in muscle thickness [18, 19]. This phenomenon was already reported after eccentric exercise [20] and interestingly the authors reported that fatigue was partly influenced by muscle swelling or oedema after this type of exercise. Moreover, the colloid osmotic pressure associated with the lymphatic drainage of extravascular proteins can lead to oedema formation by inducing imbalances in protein concentrations in the serum vs. the extravascular fluid. Moreover, the effects of altitude and extreme environmental factors on hydration can also cause oedema after MUMs. It is noteworthy that these latter mechanisms are known for being greater in the calf than in the thigh.

Therefore, the aim of the present study was to test the hypotheses that (1) the increase in circumference would be more important in the calf than in the thigh; and that (2) the increase in these circumferences would be associated with muscle force loss after a MUM.

## Methods

The study was approved by the institutional ethics committee of the University of Verona, Italy (Department of Neurological, Neuropsychological, Morphological and Motor Sciences). All of the runners and healthy volunteers provided written, voluntary, informed consent before participation, and their data were anonymised. The experiment was conducted according to the Declaration of Helsinki.

### Experimental design

The race supporting this study was the “*Tor des Géants*” 2011. This race (≈330 km, positive elevation of ≈24,000 m) around the region of Val d’Aoste in Italy is considered the world’s most challenging single-stage MUM [3].

### Participants

Twenty-five male runners were enrolled in the present study, but due to withdrawal or injuries, only 11 runners (mean values ± SEM: 43.8 ± 3.9 years old, height 174 ± 2 cm, weight 68.2 ± 2.0 kg) were tested for the measurements of neuromuscular and bioimpedance parameters: during the 2 days prior to (Courmayeur, Italy, altitude 1224 m, km 0, Pre) and approximately 1 h after the run (Courmayeur, km 330, Post). The runners’ group was compared to a control group (*n* = 8, 29.3 ± 2.0 years old, 174 ± 2 cm, 70.9 ± 3.3 kg) composed of investigators submitted to the same level of sleep deprivation. These control subjects spent long hours in standing position but did not run at all.

### Muscle fatigue assessment

We measured via transcutaneous electrical nerve stimulation in both the KE and the PF muscles the potentiated low- and high-frequency doublets evoked, respectively, at 10 Hz (PS10) and 100 Hz (PS100), as well as PS10/PS100 ratio as an index of low-frequency fatigue [21]. For further methodological details, please refer to Saugy et al. 2013 [3].

### Bioimpedance analysis

The measurement of oedema and volume of the calf and thigh was performed by the bioimpedance methods. Bioimpedance spectroscopy has been validated to measure oedema in lower limb [22]. Moreover, this method has been considered a reliable and responsive method to measure lower limb swelling [23]. In addition, the multi-frequency impedance meter used in our study (i.e. Z-métrix^®^) was validated to measure water volumes, in standing and supine position, and independently to determine total and, extra- and intracellular water compartments [24]. As previously described, we measured the circumference of the thigh and the calf with a no-stretch tape which is a suitable method to display a non-significant difference, as well as excellent correlations with the volume determined by water displacement [23]. The circumference of the thigh was measured at ¾ʺ above the patella and at 11¾ʺ above the patella. The circumference of the calf was measured at the largest part of the muscle at approximately 4″ below the patella. Dominant calf and thigh circumference were measured by the same investigators with the subject in a sitting posture for a relaxed muscle with the knee flexed to 90°. The largest circumference for both the calf and thigh were measured before the MUM and the position of the tape was marked on the skin to ensure the same location for the two other sessions. The body composition of the runners and control participants was analysed by a multi-frequency impedance meter (Z-métrix^®^, BioparHom©, Le Bourget du Lac, France). This apparatus has been validated to measure whole and segmental body composition analyses with two electrodes on the right hand and two on the right foot as previously described [24, 25]. Hydric, tissue and metabolic indicators were calculated with undisclosed equations (for more details concerning bioimpedance analyses, please see the following articles [26–29]). Total body hydration, total hydration of the non-fat mass (NF-Hyd), extracellular mass, total body water, extracellular water volume, and intracellular water volume were notably calculated in both runners and control participants. Bioimpedance parameters have been shown to be accurate and reproducible when performed with techniques using single or multi-frequency impedance meters [24–26, 28, 30]. Using bioimpedance presents advantages over the measurement of circumference, which might be only minimally increased when performed shortly after a race (a 2 % increase in calf circumference after the present MUM). Bioimpedance is non-invasive and straightforward and therefore very practical for oedema assessment.

### Statistical analysis

Two-way repeated measures analysis of variance (ANOVA) was used to identify differences in neuromuscular and bioimpedance parameters by examination of the group (runners vs. controls) × time (Pre vs. Post) interaction, complemented by Bonferroni’s post hoc test when applicable. Pearson’s correlation coefficients were calculated between Pre- and Post-MUM percentage changes in neuromuscular and bioimpedance parameters. For all statistical analyses, a *p* value of 0.05 was accepted as the level of significance (Statview 2.20; Adept Scientific, Letchworth, UK). Data were tested for equality of variance using the Fisher–Snedecor *F* test and for normality using the Shapiro–Wilk test. All data were normally distributed and are presented as the mean value ± SEM.

## Results

The runners (*n* = 11) completed the MUM in 122 ± 5 h.

### Muscle fatigue

The neuromuscular parameters are detailed in Table 1. The decrease in KE (−16.7 %) and PF (−23.4 %) PS10 values as well as in KE (−15.6 %) and PF (−21.7 %) PS100 values. In the control group, only PF PS10 and PS 100 values significantly decreased (−13.6 % for both). Significant group-by-time interactions were found only in KE PS10 (*p* = 0.0004) and PS100 (*p* = 0406).

**Table 1:** Potentiated low- and high-frequency doublets of leg muscles’ values in the runners and control participants before (Pre) and after (Post) the mountain ultra-marathon

	Runners (*n* = 11)		Control subjects (*n* = 8)	
	Pre	Post	Pre	Post
Knee extensor (KE)				
Potentiated low-frequency doublets-PS10 (N)	143 ± 11	119 ± 15**	140 ± 8	172 ± 10^†^
Potentiated high-frequency doublets-PS100 (N)	141 ± 8	119 ± 11**	157 ± 7	159 ± 11^†^
PS10/PS100	1.01 ± 0.03	0.98 ± 0.07	0.90 ± 0.07	1.08 ± 0.02
Plantar flexor (PF)				
Potentiated low-frequency doublets-PS10 (Nm)	47 ± 4	36 ± 3**	44 ± 5	38 ± 6**
Potentiated high-frequency doublets-PS100 (Nm)	46 ± 4	36 ± 2*	44 ± 9	38 ± 7**
PS10/PS100	1.03 ± 0.04	0.99 ± 0.02	1.01 ± 0.04	1.00 ± 0.05

Values are expressed as the mean ± SEM

*PS10*/*PS100* low- to high-frequency doublet peak force ratio

* *p* < 0.05, ** *p* < 0.01 for differences between Post- and Pre-values

^†^ *p* < 0.05 for differences between runners and control subjects

Differences between Post- and Pre-values

KE PS10 in control: *p* = 0.0580

KE PS100 in control: *p* = 0.7781

PF PS10 in control: *p* = 0.0085

PF PS100 in control: *p* = 0.0064

KE PS10 in runners: *p* = 0.0029

KE PS100 in runners: *p* = 0.0058

PF PS10 in runners: *p* = 0.0030

PF PS100 in runners: *p* = 0.0202

Interactions group/time

PF PS10: *p* = 0.1433/PF PS100: *p* = 0.4666

KE PS10: *p* = 0.0004/KE PS100: *p* = 0.0406

### Body composition parameters

The circumference of the calf was increased in the runners Post-MUM (Pre vs. Post: 37.3 ± 0.7 vs. 38.1 ± 0.6 cm, *p* < 0.05), but not in the control group (37.8 ± 0.8 vs. 38.0 ± 0.9 cm, ns). No significant changes were observed for the thigh circumference in the runners (45.0 ± 0.9 vs. 45.3 ± 1.1 cm, ns) or in the control subjects (47.3 ± 1.6 vs. 46.7 ± 1.7 cm, ns). No group-by-time interactions were found in calf circumference (*p* = 0.1773) and thigh circumference (*p* = 0.0847). All the other bioimpedance parameters were significantly altered after the MUM in runners (Table 2). No significant changes were found in the control group from Pre- to Post. Significant group-by-time interactions were found in total body hydration (*p* < 0.001), total hydration of the non-fat mass (*p* < 0.001), extracellular mass (*p* = 0.0045) and the Ve/Vi ratio (p = 0.0042) (Table 2).

**Table 2:** Body composition parameters of the runners and control subjects before (Pre) and after (Post) the mountain ultra-marathon

	Runners (*n* = 11)		Control (*n* = 8)	
	Pre	Post	Pre	Post
Total body hydration-Hyd (% BW)	60 ± 1	66 ± 1*** (+10 %)	62 ± 1	61 ± 1^††^ (−2 %)
Total hydration of the non-fat mass-NF-Hyd (% BW)	72 ± 1	80 ± 1*** (+11 %)	74 ± 2	73 ± 2^††^ (−1 %)
Total body water (% BW)	46 ± 1	51 ± 1*** (+11 %)	49 ± 3	48 ± 3 (−2 %)
Extracellular mass (% BW)	35 ± 1	38 ± 1* (+9 %)	34 ± 1	33 ± 1^†^ (−3 %)
Extracellular water volume-Ve (L)	21 ± 1	24 ± 1*** (+14 %)	22 ± 1	22 ± 1 (0 %)
Intracellular water volume-Vi (L)	24 ± 1	23 ± 1* (−4 %)	26 ± 1	26 ± 2 (0 %)
Ve/Vi	88 ± 2	106 ± 6* (+20 %)	86 ± 2	83 ± 1^††^ (−3 %)

Values are expressed as the mean ± SEM

Values in bracket correspond to percentage change (Post-race minus Pre-race)

% *BW* expressed in % of body weight

* *p* < 0.05, ** *p* < 0.01, *** *p* < 0.001 for differences between Post- and Pre-values

^†^ *p* < 0.05, ^††^ *p* < 0.01 for differences between runners and control participants

### Relationship between neuromuscular and body composition parameters

**Fig. 1:**
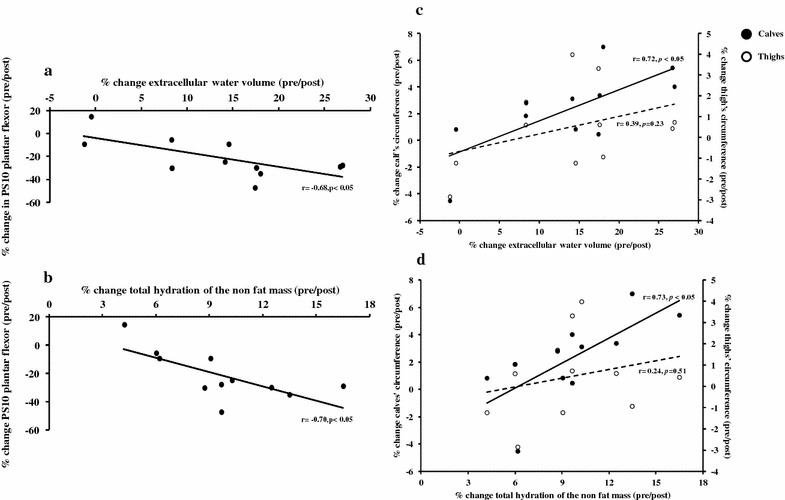
Relationships between percentage change of bioimpedance parameters and percentage change in neuromuscular parameters in runners. Pearson’s correlation coefficients were calculated between Pre- and Post-mountain ultra-marathon percentage change of bioimpedance parameters with percentage change of neuromuscular and calf and thigh circumference in runners (*n* = 11). Correlations between percentage change of extracellular water volume (Ve) and total hydration of the non-fat mass (NF-Hyd) with percentage change of potentiated low-frequency doublets of the plantar flexor (PS10) (**a**, **b**) and with percentage change of calf and thigh circumference (**c**, **d**) were calculated. The *black circles* represent data obtained from calf and the *white circles* represent data obtained from thigh. *p* value of 0.05 was accepted as the level of significance for all correlations

Negative relationships were observed between change in extracellular water volume (*r* = −0.68, *p* < 0.05) or NF-Hyd (*r* = −0.70, *p* < 0.05) and decrease in PS10 of the PF in the runners (Fig. 1a, b). The mean increase in the calf circumference was +0.8 ± 0.3 cm, +2.3 ± 0.9 % in the runners and +0.1 ± 0.2 cm, +0.3 ± 0.6 % in the control subjects. The increase in calf circumference was correlated to changes in extracellular water volume (*r* = 0.72, *p* < 0.05) or NF-Hyd (*r* = 0.73, *p* < 0.05) in the runners (Fig. 1c, d). These relationships were not observed in the thigh of the runner (mean change in the thigh circumference was +0.2 ± 0.3 cm, 0.5 ± 0.6 % in runners and −0.5 ± 0.3 cm, −1.2 ± 0.5 % in controls) (Fig. 1c, d). Finally, we did not find any relationships between neuromuscular and body composition parameters in the control group.

## Discussion

In the present study, we report that running the most challenging MUM in the world induced increases in extracellular mass, extracellular water volume and total hydration of the non-fat mass (NF-Hyd) in the runners. After the MUM, contrary to the thigh, the circumference of the calf was significantly increased in runners from Pre- to Post-. The present study showed significant relationships for the increases in total body hydration and extracellular water volume with (1) the extent of muscle force loss as quantified with PS10 in the PF (but not in the KE), and with (2) calf circumference (but not thigh circumference) between Pre- and Post-MUM. The present study suggests that increases in circumference and in hydric volume are associated to contractile impairment in the calf in ultra-marathon runners.

An increase in total body water and the development of peripheral oedema have been reported in the context of ultra-marathon running. For example, an approximately 4 % increase in total body water was reported after a 1200 km run within 17 consecutive days [12]. As expected here, we reported an increase in total body water (+10 %) in runners Post-MUM. This result could be explained by muscle tissue catabolism and increased plasma osmolality as recently reported in triathletes [31]. Moreover, an increase in plasma volume due to sodium retention [16, 17, 32] or to an increase in the total exchangeable potassium [12] has previously been reported after endurance events [16, 33, 34]. In addition, the increase in inflammation demonstrated after various MUMs [2, 3] might also explain the increase in total body water because inflammation was shown to be associated with tissue oedema [35]. The increase in total body water during ultra-marathon running could also be explained by impaired renal function and hormonal adaptations (i.e. increases in copeptin and aldosterone serum concentrations) as previously reported in ultra-marathon runners [36, 37]. Finally, other mechanisms, such as the colloid osmotic pressure associated with the lymphatic drainage of extravascular proteins, can also cause oedema through a mechanism related to imbalance in protein concentrations in the serum vs. the extravascular fluid. In addition, the effects of altitude and extreme environmental factors on hydration and subsequently on body fluid distribution, cardiac and blood pressure-related factors can also cause an increase in total body water after the MUM.

All of the studies in the field of ultra-endurance running have confirmed the high demands placed on athletes’ bodies (i.e. muscular inflammation and damage, neuromuscular fatigue) and have reported the presence of peripheral oedema, especially in the lower extremities of the body. The results of the present study were in accordance with previous studies demonstrating that the lower limbs (i.e. calf and feet) are prone to developing volume expansion after prolonged endurance [13, 14, 38] even if no significant difference was observed between the change in calf circumference from Pre- to Post-MUM between our runners and the control group. Based on these results, we could only speculate that running a single-stage MUM induces calf circumference increases as previously described in shorter events [16, 17]. This phenomenon could lead to an increase in the volume and, further, to the development of oedema. Calf circumference increases might occur because of the rise in total body water and sympathetic tone impairment (i.e. venous return impairment and increase in capillary blood pressure), which could be involved in the fluid shift into distal compartments, as previously suggested [13–15]. These later mechanisms are known for being greater in the calf than in the thigh and could explain why the increase in circumference was observed only in the calf and not in the thigh. Moreover, during motionless prolonged standing, increased hydrostatic pressure leads to increased transcapillary fluid filtration into the interstitial space of the tissues in the distal part of the lower extremities [39]. In addition, during running, the “muscle pump” counteracts this phenomenon and limits the oedema in the calf that can conversely be exacerbated after termination of exercise. Calf circumference increases could also be potentially involved in muscular force loss. Indeed, swelling was shown to increase internal fluid pressure [18, 19], leading to an increase in muscle thickness. This phenomenon has already been reported after eccentric exercise [20], but due to the delayed process of inflammatory responses, it was usually larger after 2–3 days than immediately after the exhaustive exercise [11]. In the present study, the assessment was performed only 1 h after the termination of the exercise. It is likely that the extreme duration of the MUM explains why such swelling was observed so shortly after the race. To date, the time course of neuromuscular and inflammatory responses during the days following this extreme MUM is unknown. However, during a MUM 25–40 h in duration, it was reported that several inflammatory (e.g. CRP), muscle damage (e.g. creatine kinase) and force loss parameters remained elevated at 5 days post-exercise [2]. Therefore, it is likely that the swelling and inflammatory responses would be greater a few (2–5) days later than at post-1 h. Ishikawa et al. [11] reported that architectural changes in *soleus* fascicles after exhausting eccentric fatigue were partly influenced by muscle swelling. Because no ultrasonography was performed in our study, we can presume that similar mechanisms would explain at least partly the observed relationship between swelling and force loss.

Some previous studies have suggested that muscle force loss after long duration exercises might arise from impaired excitation–contraction coupling associated with muscle damage [40] or from an inhibition of sarcoplasmic reticulum Ca^2+^ release [41]. Moreover, it has been reported that impaired Ca^2+^ handling in muscular cells [42] is responsible for low-frequency fatigue [43]. In our study, there was no significant group-by-time interaction in PF fatigue because the control subjects exhibited a large decrease in PF PS10. The 13.6 % PF force loss in controls who had the same level of sleep deprivation than the runners and spent very long hours in standing position but did not run, cannot be compared to previous literature since there is no previous study of comparison regarding the effects of extreme sleep deprivation on postural muscles. However, sleep deprivation is known to affect postural control and to reduce level of alertness: after a night of sleep deprivation, neuronal activity decreases primarily in the cortico-thalamic network. So there is a link between neurophysiological parameters and functional deficiencies. Of importance in the present control group is that the body composition parameters, including the hydric volumes and the calf diameter, did not change and that there were no relationships between calf circumference or body composition parameters and force loss. Overall, these results show that the PF force loss in the controls is likely induced by different mechanisms than those induced by the MUM in the runners.

In addition to sleep deprivation, additional mechanisms, other than those under investigation, might also be involved in muscle force loss after MUMs. Thus, it is likely that excitation–contraction coupling is involved in muscle fatigue after a MUM because we observed a reduction in the mechanical response of the PF despite a relatively preserved M wave in runners. It has been reported in rats that a series of short maximal isometric contractions of the *semimembranosus* resulted in a decrease in isometric force that was associated with swelling of sarcoplasmic reticulum of the muscle fibres [10]. Moreover, it has been reported in humans that voluntary eccentric exercise led to delayed onset muscle soreness, resulting in fibre swelling that could be involved in the decrease in muscle force after such exercise [44]. However, this latter association between muscle swelling and muscle force decrease remains unclear because another study demonstrated that the muscle damage induced by resistance exercise in active participants was not correlated with muscle activation or the force output decrease [45].

## Conclusion

In the present study, we further explored the potential relationships between neuromuscular parameters and calf circumference after a MUM. We demonstrated significant relationships of the increases in total body hydration and extracellular water volume with the percentage change of PS10 of the PF (but not of the KE) and with the percentage change in calf circumference (but not in thigh circumference) in runners. The present study suggests that increases in circumference and in hydric volume are associated to contractile impairment in the calf in ultra-marathon runners. The mechanisms are likely induced by the ultra-long running exercise and not by the extreme level of sleep deprivation.

## Study limitations

One limitation of the present study was that no assessment was performed a few days after the end of the race, when inflammation is likely maximal.

Another major limitation of the present study was that no ultrasonography of the KE or PF muscles was performed. However, it was beyond the scope of the present study to assess changes in muscle architecture or the damages induced by this MUM.

The “muscle volume” expansion might be mediated not only by increased subcutaneous tissue (which is very unlikely after 120 h of mountain running) but also by the expansion of other extracellular space or tissues. However, because no MRI measurements were performed, it was impossible to distinguish accurately the volume expansion of the different tissues.

The sample size was small. Despite this limitation, our study population was a convenience sample because of the difficulties in recruiting athletes after this type of strenuous exercise.

## Abbreviations

ANOVA: analysis of variance
CON: control subjects
KE: knee extensor
MUM: mountain ultra-marathon
NF-Hyd: hydration of the non-fat mass
PF: plantar flexor
Post: after the MUM
Pre: before the MUM
PS10: potentiated low-frequency doublet
PS100: potentiated high-frequency doublet
S.E.M: standard error of the mean
RUNNERS: ultra-marathon runners
Ve: extracellular water volume
